# Modeling Bioinspired Fish Scale Designs via a Geometric and Numerical Approach

**DOI:** 10.3390/ma14185378

**Published:** 2021-09-17

**Authors:** Ailin Chen, Komal Thind, Kahraman G. Demir, Grace X. Gu

**Affiliations:** Department of Mechanical Engineering, University of California, Berkeley, CA 94720, USA; achen87@berkeley.edu (A.C.); komalthind@berkeley.edu (K.T.); kahraman_demir@berkeley.edu (K.G.D.)

**Keywords:** bioinspired designs, dermal armor, fish scales, flexible composites, finite element analysis

## Abstract

Fish scales serve as a natural dermal armor with remarkable flexibility and puncture resistance. Through studying fish scales, researchers can replicate these properties and tune them by adjusting their design parameters to create biomimetic scales. Overlapping scales, as seen in elasmoid scales, can lead to complex interactions between each scale. These interactions are able to maintain the stiffness of the fish’s structure with improved flexibility. Hence, it is important to understand these interactions in order to design biomimetic fish scales. Modeling the flexibility of fish scales, when subject to shear loading across a substrate, requires accounting for nonlinear relations. Current studies focus on characterizing these kinematic linear and nonlinear regions but fall short in modeling the kinematic phase shift. Here, we propose an approach that will predict when the linear-to-nonlinear transition will occur, allowing for more control of the overall behavior of the fish scale structure. Using a geometric analysis of the interacting scales, we can model the flexibility at the transition point where the scales start to engage in a nonlinear manner. The validity of these geometric predictions is investigated through finite element analysis. This investigation will allow for efficient optimization of scale-like designs and can be applied to various applications.

## 1. Introduction 

Natural materials serve as a great inspiration in the development of engineering designs [1,2,3,4,5,6,7,8,9,10,11]. For instance, fish scales can provide inspiration for protective systems and designs with variable flexibility [12,13,14]. Recent studies on fish scale designs have shown that fish scales offer remarkable mechanical properties, such as resistance to penetration, while being highly flexible, lightweight structures [15,16,17,18]. The fish scale structure of interest in this study closely resembles elasmoid scales [13,19]. This type of scale is among the most commonly found fish scale types and overcomes significant tradeoffs in mechanical properties for armor design applications [20,21]. Elasmoid scales are flexible, due to their thin structure, but also have an equivalent stiffness compared to thicker scale types [22]. Elasmoid scales are able to maintain a similar puncture resistance and be more flexible than thicker scale types because of their overlapping scale feature and hierarchical structure [23,24]. The hierarchical microstructure also allows for interchangeability between flexible and stiff mechanical behavior, due to the scales’ stiff outer mineralized layer and soft inner collagen layer of the scales [20,24]. At a macroscopic level, the overlapping feature is what enables the fish to have varying stiffness and flexibility throughout its body, while providing an effective barrier against penetration. This enables the fish to have more flexibility towards the tail for locomotion and more stiffness around the vital organs for protection [25]. Areas near the vital organs are stiffer because there is an increase in overlap amongst the scales, increasing the scale interactions. The scale interactions between these overlapping scales exhibits nonlinear behaviors, and as the interactions between the scales increase, the flexural response also changes [26,27,28].

**Figure 1:**
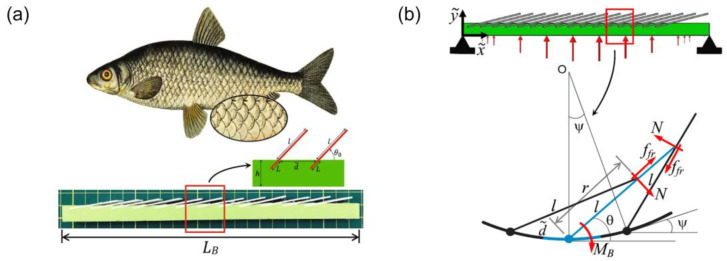
Analyzing fish scale model, adapted from reference [25]. (**a**) A 3D-printed fish scale, inspired from real fish scale. (**b**) Schematic of the fish scale interaction.

Overlapping scales are structures whose mechanical response highly depends on their configurational state at the instant of load application. The scales exhibit both linear and nonlinear behaviors, which play a significant role in the scaled design’s mechanical properties. As shown in Figure 1, the presence of scales, even before scale engagement starts, increases the stiffness of the structure [23]. Studies in literature have found that when the scales behave linearly, they do not interact with each other. In this linear region, the effect on the structure’s curvature remains minor [29]. When the scales begin interacting with each other, the structure begins to behave nonlinearly. In this nonlinear region, the scale interactions increase as the structure’s curvature increases; thus, the effect of the interactions on the mechanical response of the structure significantly increases. After analyzing different scale interaction phases, studies found that as the scale engagement increases, the structure becomes stiffer and eventually reaches a maximum curvature point where the scale structure becomes rigid [30]. Overall, these findings show that the transition point from linear-to-nonlinear, in scale behavior, plays a critical role in the stiffness and flexibility of scaled systems.

**Figure 2:**
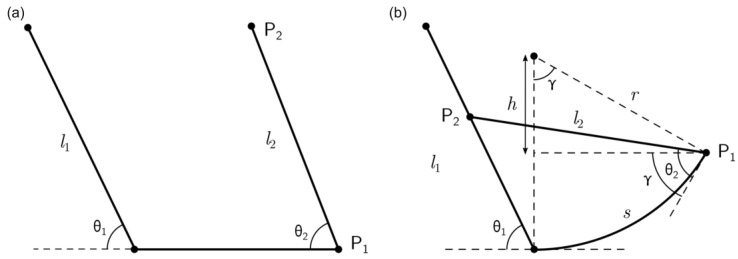
Schematic of the simplified fish scale model shown for (**a**) a neutral state and (**b**) when scales first interact.

Stiffness and flexibility are two of the most critical characteristics that measure the applicability of an armor system [13]. Current studies on fish scales focus mainly on analyzing the parameters that affect these mechanical behaviors but do not cover the methodologies that can be directly used for design applications. Using elasmoid scales as inspiration, this research focused on finding a simplistic method that can analyze the connection between the design variants of the fish scale structure and the corresponding flexibility at the transition point. In this paper, we develop a geometric model that can describe the flexibility of the fish scale model using three simple design variants. As shown in Figure 2a, the design variants of focus in this study are the spacing in between the scales, length of the scales, the angle between the scale and the hypodermis substrate. This geometric derivation takes these fish scale parameters as input and determines the structure’s flexibility, in terms of maximum curvature before scale-to-scale contact (at the transition point). To validate the accuracy of the derived equation, the fish scale model is simulated, using finite element analysis (FEA), to reproduce the interaction between scales. Advances in additive manufacturing have enabled the rapid prototyping of various materials and designs [31,32,33,34,35,36,37,38]. As a result, we have fabricated a 3D-printed fish scale model prototype, using polylactic acid (PLA) and Ninjaflex, to demonstrate the applicability of the geometric model. This study demonstrates the possibility of using a geometric model to predict the properties of the fish scale model, and its potential applicability in the design of scale-inspired flexible armor. Using this geometric model, the effect that each varying parameter has on curvature can be tested without having to manufacture each individual scale and substrate configuration.

## 2. Methods

The fish scale structure is a very complex system that constitutes multiple stiff scales inserted in a soft substrate. Each scale can have varying properties, such as size and overlapping ratio. Therefore, in this study, the effects of these variables on the fish scale model were simplified into a 2D model, as shown in Figure 2a. The model represents two scales, as two lines. We assumed the left scale was long enough that the $P_{2}$ always landed on the left scale. We also limited the distance between two scales, so that the substrate deformed with a uniform radius of curvature and the two angles $\left(\theta_{1},\theta_{2}\right)$ stayed constant. This research focused on studying the effects on the curvature of the substrate system, as the spacing, length of the scale, and the angles between the scale and substrate varies. The spacing, angle, and length were chosen as key parameters in this research, due to their dominant influence on scale-to-scale interactions, which, in return, directly affects the flexibility of the fish scale structure and needs to be taken into consideration. 

### 2.1. Geometric Model 

Firstly, we created a geometric model that could analyze the fish scales’ interaction. To tackle the complexity of interacting scales, the structure was simplified to a two-scale system. After simplification, there were a total of three design degrees-of-freedom in the geometric fish scale model. Comparatively, the only motion degree-of-freedom was the radius of curvature. As shown in Figure 2a, the parameters of importance for this study were the lengths of both scales $\left(l_{1},l_{2}\right)$, spacing in between the scales (s), and the angles of each scale, with respect to the substrate $\left(\theta_{1},\theta_{2}\right)$. After targeting the key variables, the equation could be constructed through the geometric relationship between each variable. To derive the geometric equations, the focus was on $P_{1\;}\left(x_{1},y_{1}\right)$ and $P_{2}\left(x_{2},y_{2}\right)$, as shown in Figure 2b. Since the contact point was critical in this study, the geometric equations were derived at the point where $P_{2}$ first came into contact with the left scale. The term (s) is defined as the spacing between the scales. Once force was applied to the system and the second scale started moving towards the first scale, the spacing (s) became the arc length of the curved structure. Through defining coordinates $P_{1}$ and $P_{2}$, a relationship was formed between arc length and the arc angle (γ). Using $\gamma =\frac{s}{r}$, the following geometric equation for the radius of curvature (r) was found:

(1) $$-\tan\left(\theta_{1}\right)=\frac{y_{2}}{x_{2}}=\frac{r(1-\cos\left(\gamma \right)\;)+l_{2}\sin(\theta_{2}-\gamma )}{r\sin\left(\gamma \right)-l_{2}\cos\left(\theta_{2}-\gamma \right)}\;$$

Equation (1) relates the radius of curvature to the parameters in Figure 2b (see Appendix A for the derivation of Equation (1)). By taking the inverse of this radius of curvature, the curvature of the structure was determined to get direct information about the flexibility of the scaled design. This is an important parameter in flexible armor design, for example, because if the substrate curves more, the curvature value will increase, which, in return, shows that the model is more flexible. To solve the radius of curvature from Equation (1), Taylor series was used to approximate the roots of the equation. After simplification, the above equation became a quintic polynomial, for which all the roots could be easily found using numerical approximation (see Appendix B for the simplified equations. By inputting the length, spacing, and angle values into Equation (1), the geometric model can provide an estimate of the flexibility of the fish scale design. 

### 2.2. FEA Model

**Figure 3:**
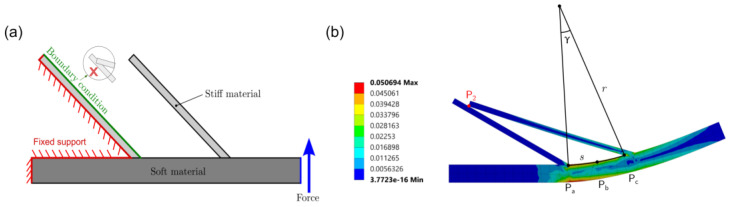
Fish scale model in FEA. (**a**) A schematic to show the FEA simulation setup including the material properties and boundary conditions. (**b**) FEA solution of equivalent strain with relevant points ($P_{a},P_{b},P_{c}$) and dimensions (γ, s) for the calculation of the radius of curvature (r). The color bar represents the elastic strain of the model.

To validate the results from the geometric equation, the fish scale structure was tested using finite element analysis in ANSYS. In order to replicate the difference in stiffness of the fish mode, the FEA was set up using two materials in Figure 3a. The substrate was set to have a Young’s modulus of 3.3 GPa and a Poisson’s ratio of 0.36, which matched the material properties of the PLA in the experiment [29,39]. The scale had a Young’s modulus of 12 MPa and a Poisson’s ratio of 0.48, matching the material properties of the Ninjaflex in the experiment [39,40]. Plane stress was used in the FEA model, and the element type of the simulation was plane183, which was a quadratic element. Additionally, the model had 882 nodes and 213 elements. As shown in Figure 3b, the left tip of the substrate was assigned to be the fixed support. By restricting a boundary condition on the first scale, the second scale would not pass through the first scale to avoid penetration. A vertical upward force was then applied at the right edge of the substrate to simulate the bending. In Figure 3b, the right edge of the first scale, the midpoint between two scales, and the left edge of the second scale on the top side of the substrate were marked as target points (labeled $P_{a},P_{b},P_{c}$, respectively) in the simulation. As the applied force increased, the displacement in both the x and the y directions of these points was recorded, to calculate the substrate’s curvature during the simulation. After knowing the initial and final positions of these three points, the curvature of the substrate created by these points could be calculated, using a perpendicular bisector theorem [41]. Assuming that the final position of $P_{a}$ was $\left(x_{1},y_{1}\right)$, $P_{b}$ was $\left(x_{2},y_{2}\right)$, and $P_{c}$ was $\left(x_{3},y_{3}\right)$, the y-intercept of the perpendicular bisector $\left(b_{1}\right)$ between $P_{1}$ and $P_{2}$ could be determined using Equation (2). Similarly, the y-intercept of the perpendicular bisector $\left(b_{2}\right)$ between $P_{2}$ and $P_{1}$ could be determined using Equation (3). After knowing the y-intercept, the center of curvature (x,y) could be calculated using Equations (4) and (5). In the end, from the position of the center of curvature, the curvature of the substrate (k) could be calculated using Equation (6).

(2) $${\left(x_{1}\right)}^{2}-{\left(x_{2}\right)}^{2}+2b_{1}\left(y_{2}-y_{1}\right)={\left(y_{2}\right)}^{2}-{\left(y_{1}\right)}^{2}$$

(3) $${\left(x_{2}\right)}^{2}-{\left(x_{3}\right)}^{2}+2b_{2}\left(y_{3}-y_{2}\right)={\left(y_{3}\right)}^{2}-{\left(y_{2}\right)}^{2}$$

(4) $$x=\frac{b_{1}-b_{2}}{\left(\frac{x_{2}-x_{3}}{y_{3}-y_{2}}\right)-\left(\frac{x_{1}-x_{2}}{y_{2}-y_{1}}\right)}\;$$

(5) $$y=\left(\frac{x_{2}-x_{3}}{y_{3}-y_{2}}\right)\;x+b_{2}\;$$

(6) $$k=\frac{1}{\sqrt{{\left(x-x_{1}\right)}^{2}+{\left(y-y_{1}\right)}^{2}}}\;$$

The y-displacement of the top end of the scale was also recorded from the simulation and used to determine the time at which the scale first came in contact. Due to the applied force, the tip of the second scale will move closer to the substrate, causing an increase in the y-displacement, until it touches the first scale. Using this phenomenon, the first scale intersection time can be determined by the maximum y-displacement of the tip. Analyzing the simulation data using Equations (2)–(6), the curvature of the substrate, when the two scales are first engaged, can be automatically calculated.

## 3. Results

### 3.1. Geometric Model Result

**Figure 4:**
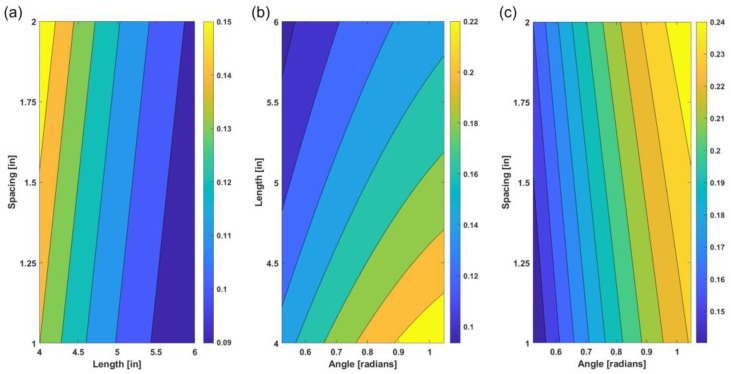
Geometric curvature trends. (**a**) The effect on curvature as the length of the second fish scale and spacing in between the scales vary. (**b**) The effect on curvature as the angle of the second fish scale and the spacing in between the scales vary. (**c**) The effect on curvature as the length of the second scale and angle of the scale varies.

After deriving the geometric equation, we validated the derived numerical method through the analysis of curvature trends and simulation results. Using the data from the derived geometric Equation (1), the contour plots analyzed how the structure’s curvature was affected by the equations’ varying parameters. As shown in Figure 4a–c, the three plots represent how the curvature changed when key parameters, such as the spacing in between the scales, the length of the second scale, and the angle of the second scale, varied. In Figure 4a, the curvature was a function of the length of the second scale and the spacing in between the scales. Here, the curvature decreased as the length of the scale increased and the curvature increased as the spacing in between the scale also increased. In addition to discovering the effects of spacing and length on curvature, Figure 4b,c further confirmed this trend and showed that increasing the angle between the scales and substrate caused an increase in curvature. The angle of the scales, with respect to the substrate, has a dominant role in the effect of curvature. Overall, these figures provide not only insight into curvature trends but also a confirmation of consistency, regarding how curvature is affected when the key parameters vary.

### 3.2. FEA Model Result

**Figure 5:**
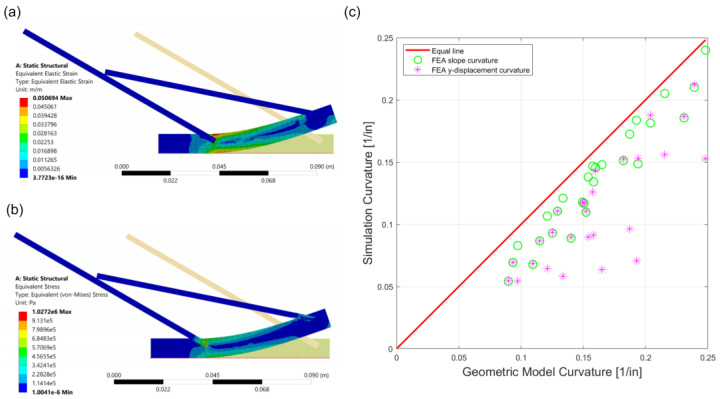
FEA simulation result. (**a**) Average von Mises strain of a fish scale model with 4-inch length, 2-inch spacing, and 30-degree angle. (**b**) Average von Mises stress of a fish scale model with 4-inch length, 2-inch spacing, and 30-degree angle. (**c**) Comparison plot between geometric and FEA results.

After investigating both the analytical and the FEA models (Figure 5a,b), the two methods were compared using a comparison plot. In Figure 5c, the *x*-axis is the curvature result from the geometric equation, and the *y*-axis represents the curvature from the finite element analysis. Inside the FEA data group, the scale lengths were 4-inch, 5-inch, and 6-inch; the spacing between the two scales varied from 1-inch, 1.5-inch, to 2-inches; and the angles varied from 30, 45, and 60 degrees. We observed that the slope of the curvature vs. force plot was relatively constant until the scales started engaging, and the slope decreased beyond the contact point. Using this discovery, a second method to automatically generate the curvature was compared with the y-displacement method in Table 1. Both methods had a percentage of error of about 10%, relative to the geometric equation, which shows the geometric equation has the ability to predict the curvature of the fish scale structure. Comparing the two curvature generating methods in FEA, the slope method was more accurate than the y-displacement method. Overall, the smallest percentages of error between the curvature results found in the slope and the y-displacement methods were 3.30% and 7.94%, respectively, which is reasonably good.

## 4. Discussion

The geometric equation provides a convenient way to predict the flexibility of the fish scale model. This equation presents a good structure property estimation of the next generation materials inspired by fish scales, without the need to model every single scenario. Three significant parameters that affect the flexibility of the model are isolated to perform an in-depth analysis of the interaction between the fish scale and its substrate. The trend of how these parameters influence the bending of the fish model is demonstrated in Figure 4a–c. As the spacing between the two scales increases, the curvature of the substrate increases; as the angle between the scale and the substrate increases, the curvature of the substrate also increases. If all other parameters are held constant but the length of the scale is increased, the curvature of the substrate decreases. This provides a useful tool for designing flexible armor systems, where the local flexibility of the system needs to be freely customized.

The plots in Figure 4 not only allow us to comprehend curvature trends, but they also serve as a verification for the derived geometric equation. The comparison plot and the representative data (Table 1) between the geometric equation result and the FEA simulation result shows the accuracy of the geometric equation. The curvature trends from both results match up completely. However, we discovered that the percentage of error grew, as the deformation of the FEA model increased. Additionally, during the simulation, material properties and the thickness of the fish scale model were considered while calculating the curvature of the substrate. Although the geometric equation simplifies the fish model and does not consider the material properties, it can correctly estimate the flexibility of the fish scale, with a small percentage of error, compared to the FEA model.

**Figure 6:**
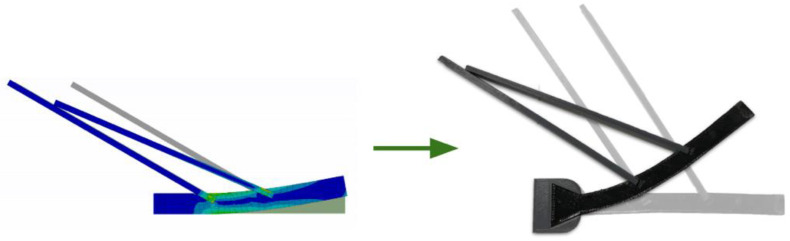
Comparing the scale interaction between the FEA model (**left**) and the 3D-printed fish scale (**right**).

To imitate the real fish scale structure’s material features, a 3D-printed fish scale sample was fabricated using PLA and Ninjaflex (Figure 6). The infill of the scale and the substrates were 100% and 50%, respectively. After printing, the scales and the substrate were assembled using super glue. This figure demonstrates the physical testing of the fish scale sample. The holding station was 3D-printed and glued on a piece of wood to provide fixed support of the fish scale during the testing. A wire was tied in a hole, located on the right edge of the substrate. When the wire was pulled, it caused the substrate to bend and the scales to interact, due to the force being applied to the sample. When modeling the bending process, using the 3D-printed sample, the interaction between the scales was the same as was predicted in the geometric equation and FEA simulation. This further demonstrates the applicability of the analytical model. The curvature of the 3D-printed model needs more investigation, due to the increasing number of varying parameters during the printing and measuring process, such as the infill percentage and infill geometry. A systematic study on the 3D-printing settings and method for finding the primary parameters (that contribute to the differences between the three methods) can improve the results.

Future work may include discovering the compatibility of the geometric equation, in which different material and printing parameters are used in the fabrication process. Finding other fish scale parameters, such as the thickness of the fish scale and the width of the 3D model, as well as applying optimization and machine learning techniques, could further enhance the accuracy of the curvature prediction [42,43,44,45,46]. On the other hand, using the geometric equation as the basic principle, a prediction tool for flexible material designs can also be developed to accommodate a more complex 3D fish scale structure.

## 5. Conclusions

Fish scales serve as a natural dermal armor with remarkable properties, such as stiffness and flexibility. By thoroughly understanding fish scale structure mechanisms, researchers can develop flexible armor without compromising stiffness. Past studies have focused on understanding fish skin mechanics, but the analysis done in this study focuses on creating a quick and effective method for design applications. By inputting parameters into the derived geometric equation, the curvature can be quantified, and one can easily get a simple assessment for their armor design. The geometric equation is derived at the transition point where the simplified two-scale system first comes into contact. This is a crucial point because it defines the transition where the scale interactions start to play a role in the design’s stiffness or flexibility. The radius of the curvature from the geometric equation is then further verified using FEA. The simulation curvature and geometric equation curvature results are close in value, with a small percentage of error (three percent). A 3D-printed sample is also presented in this study as a visual representation of the scales’ interaction in the fish scale model. By using the validated geometric equation introduced in this study, one does not have to individually manufacture and test the effect that each varying scale parameter has on curvature. This allows the designer to easily obtain a preliminary assessment, in which value is needed for the scale length, spacing, or angle, in order to have interchangeable flexibility or stiffness throughout the material design.

## Figures and Tables

**Table 1:** Comparison between geometric and FEA results using a fish scale model with a scale length of 4 inches, scale spacing of 2 inches, and angle of 30 degrees.

	Geometric Model	FEA by Slope	FEA by y-Displacement
Curvature [1/in]	0.1599	0.1457	0.1430
Percent Error [%]	-	−8.880%	−10.57%

## Data Availability

Data are available from the corresponding author upon request.

## Appendix A

$$x_{1}=r\sin\left(\gamma \right)$$

$$y_{1}=r-r\cos\left(\gamma \right)\;$$

$$\left|x_{2}-x_{1}\right|=l_{2}\cos\left(\theta_{2}-\gamma \right)\;$$

$$\left|y_{2}-y_{1}\right|=l_{2}\sin\left(\theta_{2}-\gamma \right)$$

$$x_{2}=r\sin\left(\gamma \right)-l_{2}\cos\left(\theta_{2}-\gamma \right)$$

$$y_{2}=r\left(1-\cos\left(\gamma \right)\right)+l_{2}\sin\left(\theta_{2}-\gamma \right)$$

## Appendix B

$$x=-s\tan(\theta_{1})+L_{2}\left(\tan(\theta_{1}\right)\cos(\theta_{2})-\sin(\theta_{2})$$

$$x_{1}=L_{2}\left(\tan(\theta_{1}\right)\sin(\theta_{2})+\cos(\theta_{2})-\frac{s}{2}\;$$

$$x_{2}=s\frac{\tan(\theta_{1})}{6}+L_{2}\left(\frac{\sin(\theta_{2})}{2}-\frac{\tan(\theta_{1})\cos(\theta_{2})}{2}\right)\;$$

$$x_{3}=\frac{L_{2}}{6}\left(-\tan(\theta_{1}\right)\sin\left(\theta_{2}\right)-\cos(\theta_{2}))+\frac{s}{24}\;$$

$$x_{4}=\frac{L_{2}}{24}\left(\tan(\theta_{1}\right)\cos(\theta_{2})-\sin\left(\theta_{2}\right))-s\frac{\tan(\theta_{1})}{120}\;$$

$$x_{5}=\frac{L_{2}}{120}\left(\left(\tan(\theta_{1}\right)\sin(\theta_{2}\right))+\cos(\theta_{2}))\;$$

$$x_{5}{\left(\frac{s}{r}\right)}^{5}+x_{4}{\left(\frac{s}{r}\right)}^{4}+x_{3}{\left(\frac{s}{r}\right)}^{3}+x_{2}{\left(\frac{s}{r}\right)}^{2}+x_{1}{\left(\frac{s}{r}\right)}^{2}+x_{1}\left(\frac{s}{r}\right)+x=0\;$$
